# NMFP: a non-negative matrix factorization based preselection method to increase accuracy of identifying mRNA isoforms from RNA-seq data

**DOI:** 10.1186/s12864-015-2304-8

**Published:** 2016-01-11

**Authors:** Yuting Ye, Jingyi Jessica Li

**Affiliations:** Division of Biostatistics, University of California, Berkeley, 94720 Berkeley, CA USA; Department of Statistics, 8125 Math Sciences Bldg., University of California, Los Angeles, Los Angeles, 90095-1554 CA USA; Department of Human Genetics, 695 Charles E. Young Drive South, University of California, Los Angeles, Los Angeles, 90095-7088 CA USA

**Keywords:** mRNA isoform discovery, Next-generation RNA sequencing (RNA-seq), Non-negative matrix factorization (NMF), Cufflinks, SLIDE

## Abstract

**Background:**

The advent of next-generation RNA sequencing (RNA-seq) has greatly advanced transcriptomic studies, including system-wide identification and quantification of mRNA isoforms under various biological conditions. A number of computational methods have been developed to systematically identify mRNA isoforms in a high-throughput manner from RNA-seq data. However, a common drawback of these methods is that their identified mRNA isoforms contain a high percentage of false positives, especially for genes with complex splicing structures, e.g., many exons and exon junctions.

**Results:**

We have developed a preselection method called “Non-negative Matrix Factorization Preselection” (NMFP) which is designed to improve the accuracy of computational methods in identifying mRNA isoforms from RNA-seq data. We demonstrated through simulation and real data studies that NMFP can effectively shrink the search space of isoform candidates and increase the accuracy of two mainstream computational methods, Cufflinks and SLIDE, in their identification of mRNA isoforms.

**Conclusion:**

NMFP is a useful tool to preselect mRNA isoform candidates for downstream isoform discovery methods. It can greatly reduce the number of isoform candidates while maintaining a good coverage of unknown true isoforms. Adding NMFP as an upstream step, computational methods are expected to achieve better accuracy in identifying mRNA isoforms from RNA-seq data.

**Electronic supplementary material:**

The online version of this article (doi:10.1186/s12864-015-2304-8) contains supplementary material, which is available to authorized users.

## Background

In molecular biology and transcriptomics, an important question is to understand the alternative splicing process, a key step of gene transcription in diverse species, from invertebrates to mammals. Alternative splicing generates at least two mRNA isoforms (i.e., transcripts) for one gene, and these mRNA isoforms as well as their protein products can play different roles in various biological phenomena. For example, studies have found that aberrant structures or abundance of mRNA isoforms can cause various human diseases [1, 2]. Hence, systematic identification and quantification of mRNA isoforms can greatly help understand gene regulation mechanisms for both basic biology and translational medicine. For decades, mRNA isoforms of individual genes have been accurately identified and quantified by low-throughput technologies such as cDNA cloning and qPCR. However, it was impossible to conduct this task on a system-wide scale for thousands of genes simultaneously until the invention of high-throughput technologies.

More than a decade ago, microarray technologies established a high-throughput platform for identifying and quantifying mRNA isoforms of genes with known sequences. SPACE [3, 4] is a method using non-negative matrix factorization (NMF) to predict mRNA isoforms and estimate their abundance from microarray data. NMF is a popular pattern recognition method and is distinguished from other matrix factorization methods by its use of non-negativity constraints [5]. A main advantage of NMF is its interpretable factorization results, which are positive and sparse combinations of features. In the context of mRNA isoform discovery, SPACE uses NMF to find isoforms as combinations of exons and exon junctions. SPACE was demonstrated to have good power in detecting mRNA isoforms under ideal simulation settings. However, many open questions remain about how to use NMF to accurately find mRNA isoforms from real microarray data. Reasons for these questions include the following issues inherent with NMF and microarray. First, in many scenarios NMF outputs non-unique factorization results [6], making SPACE identify ambiguous mRNA isoforms. Second, NMF requires a pre-specified matrix rank for factorization results, and how to determine this rank remains a difficult question [7–9]. Third, microarray can only detect exons and exon junctions with at least partially known sequences, and thus it cannot provide information on novel exons or exon junctions. This hinders SPACE from discovering novel isoforms that contain novel exons or exon junctions.

Compared to microarray, the more recent next-generation RNA sequencing (RNA-seq) technologies yield deeper and wider insights into transcriptomes at base level resolution [10]. RNA-seq measures the expression of genome-wide exons and exon junctions, regardless of prior knowledge on their existence. Hence, unlike microarray, RNA-seq enables the identification of novel mRNA isoforms containing previously unknown exons or exon junctions. Despite its many advantages, a major drawback of Illumina RNA-seq, the most popular RNA-seq platform, is that it can only generate RNA-seq reads with short lengths (≤2×250 bp for paired-end reads). Such short RNA-seq reads cannot cover a full-length mRNA isoform, making isoform identification a difficult computational problem. Since the advent of RNA-seq, various computational methods have been developed to discover mRNA isoforms from RNA-seq data. For the species with good-quality reference genomes, including human and model organisms, mainstream isoform discovery methods (e.g., Cufflinks [11], Scripture [12], IsoLasso [13] and SLIDE [14]) use RNA-seq reads first mapped to a reference genome. In this paper, our discussion focuses on these so-called “refernecebased methods”, which attempt to identify full-length mRNA isoforms from mapped RNA-seq reads using various approaches. For example, Cufflinks, Scripture and IsoLasso first build graphs of exonic regions containing mapped reads, and then find mRNA isoforms as the maximal paths in the graph based on a parsimony assumption. These three methods are also often referred to as “*de novo* methods,” because their isoform identification approach can completely rely on RNA-seq reads without using existing annotations. Another reference-based method SLIDE uses a different approach. It constructs a search space of possible isoforms by using known boundaries of genes and exons from existing annotations, with the possible addition of novel genes and exons inferred from RNA-seq data. SLIDE then identifies isoforms from the search space based on a regularized linear model, which accounts for the RNA-seq read generating mechanism and read distributions. Because of its use of annotations, SLIDE is also called an “annotation aided method”, in contrast to the previous “*de novo* methods.” Note that the recent versions of Cufflinks also added an annotation aided option, which can simulate RNA-seq reads from annotations and use the simulated reads together with RNA-seq data for isoform discovery [15]. Both *de novo* and annotation aided methods have advantages and drawbacks. The graph-based *de novo* methods are more robust to RNA-seq data noise thanks to their use of the parsimony assumption, which, however, also prevents them from finding overlapping isoforms. SLIDE, on the other hand, is capable of finding overlapping isoforms but is more sensitive to RNA-seq data noise. Despite their differences, a common obstacle for both types of methods is the large search space of possible mRNA isoforms for genes with complex splicing structures (i.e., many exons). For the graph-based *de novo* methods, a gene with many exons will lead to a graph with large numbers of nodes and edges, making it difficult to search for isoforms as maximal paths. For SLIDE, a gene with *n* exons will give rise to a total of 2^*n*^−1 possible isoforms, placing great difficulty on the regularized linear model to find the correct isoforms. Although SLIDE is coupled with a naïve preselection procedure to filter out the isoform candidates with unsupported exon junctions by RNA-seq reads, this procedure is not ideal for lowly expressed isoforms, whose exon junctions may not be supported by reads due to the low read coverage.

Given the wide existence of RNA-seq noise and the large search space of possible isoforms, existing computational methods for isoform discovery have unsatisfactory performance in many cases. In an evaluation conducted by the RGASP consortium [16], Cufflinks only achieved precision rates of 23 %, 50 % and 47 % and recall rates of 18 %, 40 % and 39 % for discovering full-length mRNA isoforms from *H. sapiens*, *D. melanogaster*, and *C. elegans* RNA-seq data respectively. For SLIDE, the corresponding precision rates were 37 %, 80 % and 63 %, and the corresponding recall rates were 7 %, 49 % and 59 %. This evaluation showed that both Cufflinks and SLIDE, as well as other evaluated methods, have low accuracy for discovering human isoforms, mainly because many human genes have large numbers of exons and thus a huge search space of possible isoforms. In statistical literature, for high-dimensional linear models with far more features than observations, it was found that adding a screening procedure to filter out excessive features in the search space can improve the feature identification performance of Lasso [17]. Since Lasso was used in SLIDE to find isoforms as features, we are motivated to reduce the search space of possible isoforms by designing a screening (or preselection) procedure, so as to improve the performance of SLIDE and hopefully other isoform identification methods.

Also motivated by SPACE and the good properties of NMF, we propose a preselection method “Non-negative Matrix Factorization Preselection” (NMFP) to improve the accuracy of reference-based isoform discovery methods. Our goal is to use NMFP to greatly reduce the search space of isoforms, and have the selected isoform candidates cover most of the true isoforms. NMFP employs NMF to select isoform candidates for a given gene by decomposing its RNA-seq read count matrix. We demonstrate the performance of NMFP by simulation and real data studies. In the simulation, we generate RNA-seq reads from *D. melanogaster* (fly) and *M. musculus* (mouse) using Flux Simulator [18] and show that NMFP can greatly improve the accuracy of Cufflinks and SLIDE in isoform discovery. We also show the efficacy of NMFP on discovering *D. melanogaster* isoforms from real RNA-seq data. Moreover, we evaluate the robustness of NMFP to the choice of NMF ranks, the number of input samples in the read count matrix, and gene expression levels. In addition, we show that NMFP can increase the robustness of SLIDE to its regularization parameter choice.

## Methods

Our proposed method NMFP shares the same root as SPACE, because they both use NMF to identify mRNA isoforms. However, unlike SPACE that aims to directly identify isoforms via NMF, NMFP has a less ambitious goal, which is to identify a set of good isoform candidates for downstream isoform discovery methods. To achieve this goal, in NMFP we use high-frequency filtering to alleviate the non-uniqueness issue of NMF results, and design a model selection approach to choose the NMF rank. Moreover, we develop a new NMF algorithm to penalize the isoforms that have conflicting exons or exon junctions.

### Data processing

Suppose there are *m* RNA-seq data sets (samples) and a gene has *nsubexons*, which were defined as the exonic regions between two adjacent splicing sites in SLIDE [14]. For this gene, we first categorize RNA-seq reads in every sample into *bins*, which are defined as two-dimensional vectors (*k, l*), representing the mapped reads (single-end reads or two ends of paired-end reads) with starting position in the *k*-th subexon and ending position in the *l*-th subexon, 1≤*k*≤*l*≤*n*. Hence, this gene has a total number of $p = n + {n \choose 2}$ bins. We count the numbers of reads in every bin in all samples and generate a read count matrix **U** with dimensions *p*×*m*, with rows corresponding to bins and columns corresponding to samples

Since samples may have different sequencing depths and bins may have different lengths (due to different lengths of exons), to prevent the NMF results from being dominated by samples with more reads or bins with greater lengths, we normalize **U** into a matrix **V** by removing the sequencing depth and bin length effects. We conduct the normalization based on the following model

(1) $$ U_{ij}=R_{j} \cdot P_{i}~,  $$

where *U*_*ij*_ is the (*i,j*)-th entry of **U**, representing the read count of the *i*-th bin in the *j*-th sample, *R*_*j*_ is the total number of reads mapped to this gene in the *j*-th sample, and *P*_*i*_ is the probability that a read falls into the *i*-th bin (0≤*P*_*i*_≤1 and $\sum _{i=1}^{p} P_{i}=1$). We assume that *P*_*i*_ is proportional to the length of the *i*-th bin. Although this assumption can be violated by the non-uniformity of read distribution due to alternative splicing and sequencing noise, it provides an approximate and simple solution to removing bin length effects from the read count matrix.

We describe the normalization process as follows.

1. Scale the read counts of each sample inverse proportionally with its total number of reads
  $$U'_{ij}=U_{ij} \cdot \frac{\bar{R}}{R_{j}}~,~i=1,2,\ldots,p~;~j=1,2,\ldots,m~, $$
  where $\bar {R}=\frac {1}{m}\sum _{j=1}^{m} R_{j}~$.
2. Further scale the read counts of each bin inverse proportionally with its bin length
  $$V_{ij}=U'_{ij} \cdot \frac{1}{p P_{i}}~,~i=1,2,\ldots,p~;~j=1,2,\ldots,m~, $$
  and denote **V**=(*V*_*ij*_), where *V*_*ij*_ represents the normalized read count of the *i*-th bin in the *j*-th sample.

### Matrix decomposition model

Li and Wong [19] proposed a model to describe the relationship between the probe intensity *v* and the transcript abundance *t* for microarray data

(2) $$ v=a \cdot t+e~,   $$

where *a* is the affinity of the probe, and *e* is an error term. SPACE revised the Model (2) into the following matrix model for *p* probes and *s* transcripts

(3) $$ \textbf{V} = \textbf{A} \cdot \textbf{G} \cdot \textbf{T} + \boldsymbol{\epsilon}~,  $$

where **V** is a *p*×*m* matrix representing the intensities of *p* probes in *m* samples; **A** is a *p*×*p* diagonal matrix representing the affinity of *p* probes; G is a *p*×*s* indicator matrix with binary elements indicating the existence of the *p* probes in *s* transcripts; **T** is an *s*×*m* matrix representing the abundance of *s* transcripts in *m* samples; ***ε*** is a *p*×*m* error matrix.

Inspired by the Models (2) and (3), we write the following model for the relationship between normalized reads counts and unknown transcript (i.e., mRNA isoform) abundance

(4) $$ \textbf{V} = \textbf{W} \cdot \textbf{H} + \boldsymbol{\epsilon}~,  $$

where **V** is the *p*×*m* normalized reads count matrix (from last section “Data processing”), and **W** is a *p*×*s* isoform composition matrix, whose rows and columns are bins and mRNA isoforms respectively. Each column of **W** is a *p*-dimensional vector with elements ∈ [ 0,1], indicating the existence of bins in an isoform, with the values subject to RNA-seq noise and biases. Theoretically, all the existing bins in an isoform should have the same read density assuming that RNA-seq reads follow a uniform distribution in that isoform. However, due to RNA-seq noise and biases, the uniformity assumption is often violated, and non-existing bins may contain erroneously mapped reads. To account for these issues, we model the existence of bins in an isoform as values between 0 and 1, where 1 indicates a bin having the maximal read intensity among all the bins, and 0 indicates a bin containing no mapped reads. **H** is a *s*×*m* isoform abundance matrix, whose rows and columns are mRNA isoforms and samples respectively. Each row of **H** is an *m*-dimensional vector representing the isoform’s abundance levels in the *m* samples. Based on our definition of **W**, *h*_*ij*_ (the abundance value of the *i*-th isoform in the *j*-th sample) should be equal to the maximum bin abundance of the *i*-th isoform in the *j*-th sample. ***ε*** is an error matrix.

We can further decompose **W** as

(5) $$ \textbf{W} = \textbf{A} \cdot \textbf{G}  $$

where **A** is a *p*×*p* diagonal matrix with elements ∈ [ 0,1], representing the biases of read counts in different bins. Such biases are attributable to the features of the bins, such as GC contents and the bins’ positions in transcripts. **G** is a *p*×*s* indicator matrix with binary values to describe the existence of bins in isoforms. Each column of **G** represents an isoform.

### Modified NMF approach

With the normalized read count matrix **V**, our goal is to estimate **G**, whose columns will be regarded as isoform candidates. To achieve this goal, we have to first estimate **W** and **A**. We adopt the SPACE approach to estimate **G**. In the SPACE method [4], given an estimate $\hat {\textbf {W}}=(\hat {W}_{\textit {ik}})$, **A** was estimated as

(6) $$ {}\hat{\textbf{A}} = \text{diag}\!\left(\hat{A}_{11}, \ldots, \hat{A}_{pp}\right),~\text{where}\,\, \hat{A}_{ii}=\max\limits_{k=1,\cdots,s}({\hat{W}_{ik}})~.  $$

In other words, $\hat {A}_{\textit {ii}}$, the bias factor of read counts in the *i*-th bin, is considered as $\max _{k} (\hat {W}_{\textit {ik}})$, the largest “adjusted existence” of the *i*-th bin in all isoforms.

With $\hat {\mathbf A}$ and $\hat {\mathbf W}$, **G** is estimated as

(7) $$ \hat{\mathbf{G}}=(\hat{G}_{ij}),\ \text{with}\ \hat{G}_{ij} = I\left((\hat{\mathbf{A}}^{-1}\cdot \hat{\mathbf{W}})_{ij} \ge c\right),   $$

where *I*(·) is an indicator function, and *c*∈IR is a threshold to make $\hat {\mathbf {G}}$ a binary matrix. We used *c*=0.4 in this paper.

Prior to that, we estimate **W** by decomposing **V** using NMF. There exist many available NMF algorithms [5, 20–22], which have different preferences over the factorization results. Since our goal is to find isoform candidates by decomposing **V**, one constraint we need is the compatibility of bins in every column of $\hat {\textbf {W}}$, i.e., every isoform found by NMF. For example, Bin (1,3) and Bin (2,2) are conflicting and should not co-exist in an isoform, because the former indicates that Subexon 2 is skipped, while the latter implies the existence of Subexon 2.

To realize this constraint in NMF, we add a penalty term to the standard NMF objective function as follows

(8) $$ \begin{aligned} {}D(\textbf{V,\, W H})=&\sum\limits_{i=1}^{p}\sum\limits_{j=1}^{m} \left(V_{ij} \log \frac{V_{ij}}{(\mathbf{WH})_{ij}}-V_{ij}+ (\mathbf{WH})_{ij} \right)\\ &+\alpha\sum\limits_{i \perp j}\left(\textbf{WW}^{T}\right)_{ij}\\ \end{aligned}  $$

where *p* is the number of bins, *m* is the number of samples, and *α* is a regularization parameter controlling the weight of the penalty term (e.g., *α*=0.1). *i*⊥*j* denotes that Bin *i* and Bin *j* are conflicting (See Section 1 in the Additional file 1 for more detail). We estimate **W** by minimizing the objective function *D*(**V, WH**) subject to a pre-specified *s*=rank(**W**), which is equivalent to the number of columns of **W**. How to determine the rank remains an open question. In NMFP, we choose the rank by gap statistic, which was originally designed to select the number of clusters in *k*-means clustering [23] (See Section 2 in the Additional file 1 for more detail). After obtaining $\hat {\textbf {W}}$, we use Eqs. (6) and (7) to find $\hat {\textbf {G}}$, which indicates a set of isoform candidates.

The penalty term in the objective function (8) cannot fully prevent the co-existence of conflicting bins in every isoform (i.e, column in $\hat {\textbf {G}}$). To resolve this issue and recover potentially true isoforms, we consider both the existing and skipped statuses of the ambiguous subexons that lead to conflicting bins. For example, if both Bin (1,3) and Bin (2,2) exist, Subexon 2 is ambiguous. We will consider the cases of Subexon 2 being existing and skipped as two different isoforms. If there are *n* ambiguous subexons in an isoform candidate, 2^*n*^ isforms will be considered, but only those whose subexons and subexon junctions are supported by RNA-seq reads will be kept as isoform candidates.

### Aggregation of multiple NMF runs

To address the issue of non-unique NMF results, we use a high-frequency filtering approach to find isoform candidates identified in a number of NMF runs. Specifically, we implement the NMF estimation procedure described in the previous two subsections for *N* times (e.g. *N*=100) and aggregated the identified isoform candidates into a candidate pool. In the filtering step, we first remove the isoform candidates, which contain subexon junctions not supported by reads, from the pool. Then among the rest of candidates in the pool, we only retain the high-frequency ones, which are selected in at least *r* NMF runs (e.g., *r*=20), as final isoform candidates.

**Fig. 1:**
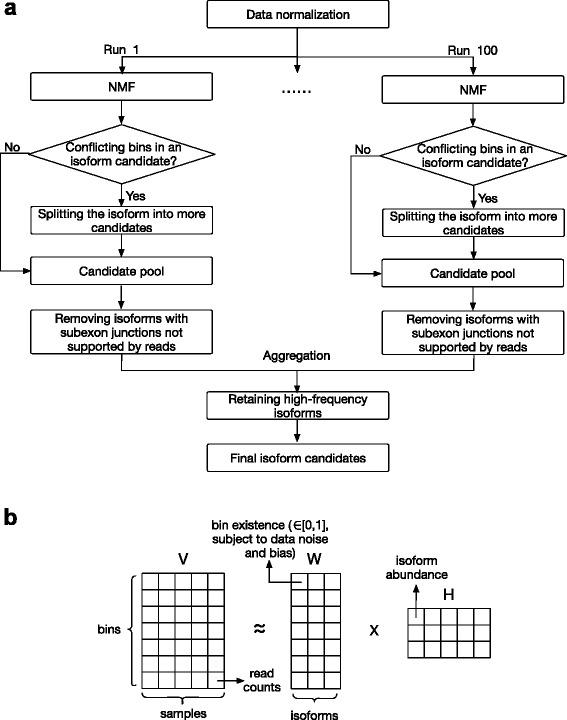
**a** Diagram of the NMFP method. **b** Illustration of the NMF approach in NMFP

The NMFP method is illustrated in Fig. 1.

## Results

We conduct simulations and real-data analyses in many scenarios to demonstrate the effectiveness of NMFP in improving the performance of downstream isoform discovery methods. The following results show that NMFP can help Cufflinks and SLIDE achieve better isoform discovery accuracy. We also study the robustness of NMFP to the choice of NMF rank, the number of input samples in the read count matrix, and the gene expression levels.

### Simulation results in *D. melanogaster*

We use Flux Simulator [18] to simulate RNA-seq reads of *D. melanogaster* (with reference genome dm6 and Ensembl annotation BDGP6 of release 80). We focus on 4125 genes on chr3R, in accordance with the SLIDE paper [14], which showed good performance of SLIDE on genes with 3 to 10 subexons. We simulate 50 samples, each with 10,000,000 RNA molecules and 50,000,000 paired-end reads with length 2×76 bp, from these genes’ isoforms in the annotation. The isoform abundance is randomly assigned by Flux Simulator. Based on the way we define subexons (Additional file 1: Figure S1), 51.7 *%* (2132) genes contain 3 to 10 subexons (Table 1). Among these genes, 44.6 *%* (951) have more than one isoforms in the annotation. We apply NMFP to these 951 genes.

**Table 1:** Profiles of 4125 genes in chr3R of *D. melanogaster*

# of subexons *n*	*n*≤2	3≤*n*≤10	11≤*n*≤15	16≤*n*≤20	*n*>20	Sum
	1618 (39.2 *%*)	2132 (51.7 *%*)	232 (5.6 *%*)	79 (1.9 *%*)	64 (1.6 *%*)	4125
# of annotated isoforms *q*	*q*=1	*q*=2	*q*=3	*q*=4	*q*>4	Sum
	2450 (59.4 *%*)	878 (21.3 *%*)	356 (8.6 *%*)	180 (4.4 *%*)	261 (6.3 *%*)	4125

To check the effects of NMFP on improving the isoform discovery performance of SLIDE, we define a new procedure called “NMFP + SLIDE”, which combines NMFP and SLIDE by inputting the isoform candidates found by NMFP into SLIDE. In the original SLIDE method, 2^*n*^−1 possible isoforms are enumerated for an *n*-subexon gene, and SLIDE uses a simple preselection to filter out the isoforms with subexons or subexon junctions not supported by reads. NMFP + SLIDE replaces this preselection step by NMFP.

Similarly, we define “NMFP + Cufflinks”, which inputs the isoform candidates found by NMFP as a GTF file into Cufflinks and uses Cufflinks with the “–GTF” option to estimate the abundance of these candidates. The candidates with non-zero estimated abundance are kept as the isoforms identified by NMFP + Cufflinks. This is different from the original Cufflinks, because it does not rely on a connectivity graph or the parsimony assumption as Cufflinks does.

To compare SLIDE with NMFP + SLIDE, and Cufflinks with NMFP + Cufflinks, we evaluate their isoform discovery performance at three different levels: the nucleotide, exon and transcript levels, similar to the RGASP evaluation [16]. In this comparison, we also evaluate the performance of NMFP alone, because it is important to check whether the isoform candidates found by NMFP have a good coverage of the true isoforms. Since SLIDE has two default values for its regularization parameter *λ* in the Lasso regression (*λ*=0.2 for fewer isoforms and *λ*=0.01 for more isoforms), we include two versions of SLIDE in this comparison: “SLIDE(fewer)” for *λ*=0.2 and “SLIDE(more)” for *λ*=0.01. This results in a comparison of seven methods in total:

- Cufflinks
- NMFP + Cufflinks
- SLIDE(fewer)
- NMFP + SLIDE(fewer)
- SLIDE(more)
- NMFP + SLIDE(more)
- NMFP

#### Evaluation at nucleotide level

We first compare the seven methods at the nucleotide level, that is, how many nucleotides are shared by the identified isoforms of each method and the true isoforms in the annotation. For each identified isoform, we match it to the annotated isoform that shares the most nucleotides with it. We rank the identified isoforms by their percentages of overlapping nucleotides with their matched annotated isoforms, from the highest percentage to the lowest. If there are *k* identified isoforms and *r* annotated isoforms and *k*>*r*, only the top *r* identified isoforms would be paired with an annotated isoform, and the rest *k*−*r* identified isoforms would be counted as false positives with zero precision rates. For each identified isoform paired with an annotated isoform, we calculate its precision rate as $\frac {\#\text {overlapping nucleotides with the paired annotated isoform}}{\# \text {nucleotides in the identified isoform}}$. The precision rate of a gene was defined as the average of the individual precision rates of its identified isoforms.

Similarly, we match each annotated isoform with an identified isoform. We calculate the recall rate of each annotated isoform with a paired identified isoform as $\frac {\#\, \text {overlapping nucleotides with the paired identified isoform}}{\#\, \text {nucleotides in the annotated isoform}}$. If there are more annotated isoforms than identified ones, the unpaired annotated isoforms would have zero recall rates. For a given gene, its recall rate is defined as the average of the individual recall rates of its annotated isoforms. Given the precision and recall rates of a gene, we calculate the F score as the their harmonic mean. That is, F score $= \frac {2}{1/\text {precision}+1/\text {recall}}$.

**Fig. 2:**
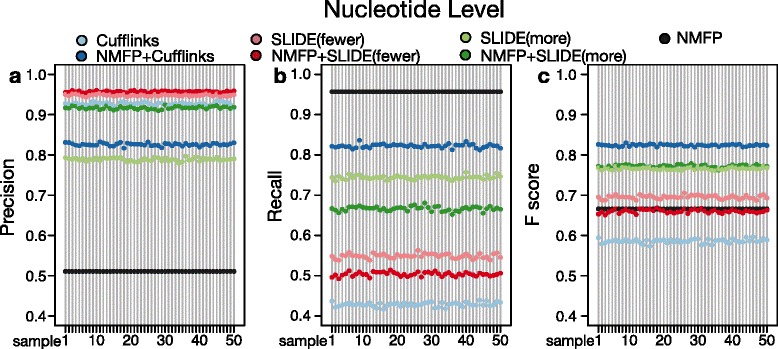
Isoform discovery performance at the nucleotide level. **a** Precision rates, **b** Recall rates, and **c** F scores of the identified isoforms by seven methods (Cufflinks, NMFP + Cufflinks, SLIDE(fewer), NMFP + SLIDE(fewer), SLIDE(more), NMFP + SLIDE(more), and NMFP) on 50 simulated RNA-seq data sets of *D. melanogaster*

We calculate the precision and recall rates of every method in each of the 50 samples by taking the average of 951 genes. In the results, all methods except NMFP have precision rates above 0.76 in all samples. Although NMFP has low precision rates, its recall rates are as high as 0.96, implying that its identified isoform candidates has a good coverage of annotated isoforms (Fig. 2). NMFP + Cufflinks has lower precision rates but much higher recall rates than Cufflinks, because the latter identifies fewer isoforms given its parsimony assumption. SLIDE has opposite results, which show that NMFP + SLIDE have higher precision rates but lower recall rates than SLIDE, for both SLIDE(fewer) and SLIDE(more). The reason is that the original versions of SLIDE have a larger search space than NMFP + SLIDE, and thus they are likely to find more isoforms and thus cover more nucleotides given the same Lasso regularization parameter.

In terms of F scores, which combine precision and recall rates, NMFP is shown to have greatly improved the performance of Cufflinks, by increasing the F scores of approximately 0.6 for Cufflinks to more than 0.8 for NMFP + Cufflinks. Strikingly, with the addition of NMFP, Cufflinks outperforms SLIDE, which originally had better F scores than Cufflinks without NMFP [14]. NMFP + Cufflinks becomes the top performer in F scores. On the other hand, NMFP does not much improve the performance SLIDE(more) and even slightly deproves the performance SLIDE(fewer) at the nucleotide level. The reason is that the original SLIDE has reasonably good performance at the nucleotide level, though its identified isoforms are often similar to but not exactly the annotated isoforms (see the subsection “Evaluation at transcript level”).

#### Evaluation at exon level

We next evaluate the performance of these seven methods at the exon level. If an exon in an identified isoform overlaps at least 50 *%* of an annotated exon, we call the two exons “overlapping”. Similar to our matching scheme at the nucleotide level, we match identified isoforms with annotated isoforms at the exon level. Then for each identified isoform, its precision rate at the exon level is defined as $\frac {\#\,\text {overlapping exons with the paired annotated isoform}}{\#\,\text {exons in the identified isoform}}$. Similarly for each annotated isoform, its recall rate is defined as $\frac {\#\,\text {overlapping exons with the paired identified isoform}}{\#\,\text {exons in the annotated isoform}}$. Then for every gene, its precision and recall rates are calculated as the average precision rate of its identified isoforms and the average recall rate of its annotated isoforms. Its F score is the harmonic mean of its precision and recall rates. For every method in every sample, the precision, recall and F score are calculated by taking the average of 951 genes.

**Fig. 3:**
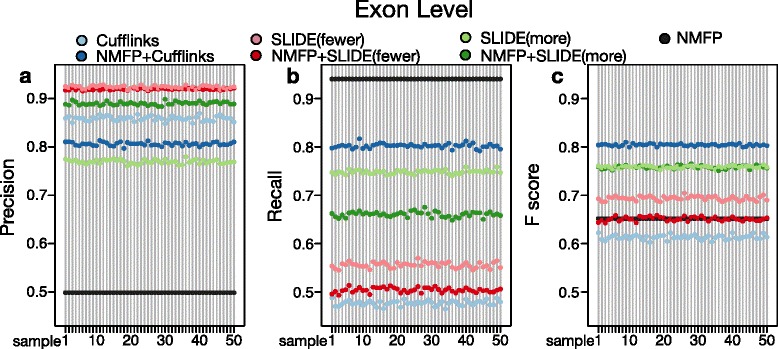
Isoform discovery performance at the exon level. **a** Precision rates, **b** Recall rates, and **c** F scores of the identified isoforms by seven methods (Cufflinks, NMFP + Cufflinks, SLIDE(fewer), NMFP + SLIDE(fewer), SLIDE(more), NMFP + SLIDE(more), and NMFP) on 50 simulated RNA-seq data sets of *D. melanogaster*

Similar to the performance at nucleotide level, NMFP significantly improves the F scores of Cufflinks from approximately 0.6 to over 0.8, yet it does not improve the performance SLIDE (Fig. 3). The reason is that the original SLIDE has reasonably good performance at the exon level, though its identified isoforms are often similar to but not exactly the annotated isoforms (see the subsection “Evaluation at transcript level”).

#### Evaluation at transcript level

Compared to the previous evaluation at nucleotide and exon levels, the evaluation at the transcript level is more important, as the ultimate goal of isoform discovery methods is to correctly identify full-length mRNA isoforms. At the transcript level, if an identified isoform and an annotated isoform have every exon matched by the criterion we defined for matching at the exon level, we called these two isoforms “matched”. Then for every gene, its precision and recall rates at the transcript level are defined as $\frac {\#\,\text {identified isoforms matched with annotated isoforms}}{\#\,\text {identified isoform}}$ and $\frac {\#\,\text {annotated isoforms matched with identified isoforms}}{\#\,\text {annotated isoform}}$ respectively. Its F-score is the harmonic mean of its precision and recall rates. For every method in every sample, the precision, recall and F score are calculated by taking the average of 951 genes.

**Fig. 4:**
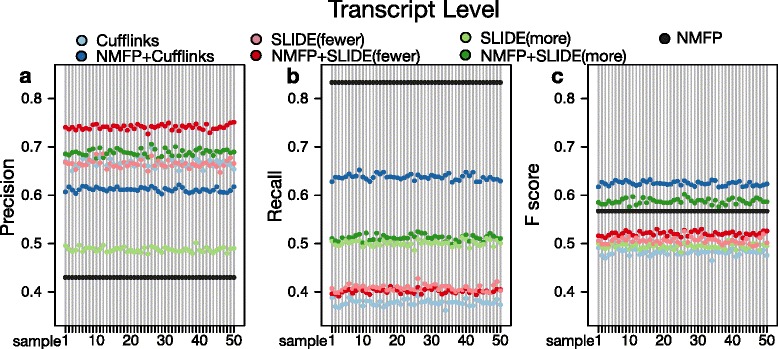
Isoform discovery performance at the transcript level. **a** Precision rates, **b** Recall rates, and **c** F scores of the identified isoforms by seven methods (Cufflinks, NMFP + Cufflinks, SLIDE(fewer), NMFP + SLIDE(fewer), SLIDE(more), NMFP + SLIDE(more), and NMFP) on 50 simulated RNA-seq data sets of *D. melanogaster*

In the results, NMFP has high recall rates greater than 0.85, indicating that the isoform candidates found by NMFP have a good coverage of annotated transcripts (Fig. 4). Meanwhile, NMFP has precision rates above 0.4, implying that the number of isoform candidates is less than 2.5 times the number of annotated isoform. This demonstrates the effectiveness of NMFP in reducing the search space of possible isoforms. In terms of F scores at the transcript level, NMFP greatly improves the performance of both Cufflinks and SLIDE. Specifically, NMFP + Cufflinks has F scores above 0.6, while Cufflinks has F scores only below 0.5. The reason is that NMFP greatly improves the recall rates of Cufflinks. NMFP also increases the F scores of SLIDE(more) from 0.5 to approximately 0.6, because NMFP helps SLIDE(more) achieve better precision rates. These results illustrate the effectiveness of NMFP in reducing the search space for SLIDE (thus increasing the precision of SLIDE) and in removing the parsimony assumption for Cufflinks (thus increasing the recall of Cufflinks). To explain why NMFP can improve SLIDE’s performance at the transcript level but not at the nucleotide or exon level, we find the reason as the unidentifiability issue of SLIDE with a large search space of possible isoforms. Due to the short RNA-seq read lengths, there may exist multiple sets of isoforms (as combinations of exons) that can well explain the observed RNA-seq reads. Hence with a large search space of isoforms, SLIDE are likely to miss the true isoforms, but it still can detect nucleotides and exons reasonably well. Since NMFP can shrink the search space to have a higher concentration of true isoforms (i.e., annotated isoforms in the simulation), SLIDE would become more likely to identify the true isoforms and thus achieve better isoform discovery accuracy at the transcript level.

#### Robustness of NMFP to the choice of NMF rank

**Fig. 5:**
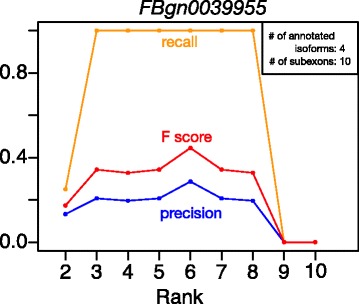
Robustness of NMFP performance to NMF rank choices. Precision (*blue*), recall (*orange*) and F scores (*red*) of the identified isoform candidates by NMFP are evaluated at 9 different NMF ranks on simulated data from *D. melanogaster* gene *FBgn0039955*, which has 10 subexons and 4 annotated isoforms

How to determine the matrix rank of factorization results is a difficult and open question in NMF [3, 4, 7–9]. In NMFP, the NMF rank determines the number of isoform candidates to be found after each NMF run. Our aggregation of multiple NMF runs and the subsequent high-frequency filtering can increase the robustness of NMFP to the choices of NMF rank. To illustrate this point, we use *D. melanogaster* gene *FBgn0039955* as an example and apply NMFP with different NMF ranks to the simulated RNA-seq data. This gene has 4 annotated isoforms (in Ensembl BDGP6 of release 80) and 10 subexons. In the result (Fig. 5), NMFP has a good recall rate of 0.9 for ranks ranging from 3 to 9. The precision rate and F score are also stable in this range. This result and other examples in the Additional file 1 show the robustness of NMFP to the choices of NMF rank. In practice, NMFP selects the rank based on gap statistic (See Section 2 in the Additional file 1 for more detail). Nevertheless, we note that the NMF rank cannot be too much smaller or larger than the number of true isoforms. If too small, the rank will lead to too few isoform candidates to cover the true isoforms. If too large, the identified isoform candidates will be mostly individual bins but not full-length isoforms.

### Simulation results in *M. musculus*

We also evaluate the performance of NMFP in *M. musculus* (mouse) (reference genome mm10 and annotation GRCm38 of release 81), a mammalian model organism with more complex gene structures than *D. melanogaster*. We generate 100 RNA-seq samples from mouse chr1 using Flux Simulator, with paired-end reads of length 2×76 bp. The 100 samples have 10 different numbers of RNA molecules and 10 different numbers of reads. The numbers of RNA molecules range from 4,200,000 to 6,000,000, increasing in steps of 200,000. The number of reads range from 11,000,000 to 20,000,000, increasing in steps of 1,000,000. More detail on the 100 samples is summarized in Section 3.2 and Table S1 of the Additional file 1. On chr1, there are 3432 genes, among which we are interested in 852 genes with 3-10 subexons and 2-17 annotated isoforms (more detail in Additional file 1: Table S2). In this simulation, we calculate precision and recall rates and F scores at the transcript level for every method, same as what we did in the simulation in *D. melanogaster*.

#### Increased robustness of NMFP + SLIDE to the choices of parameter *λ*

**Fig. 6:**
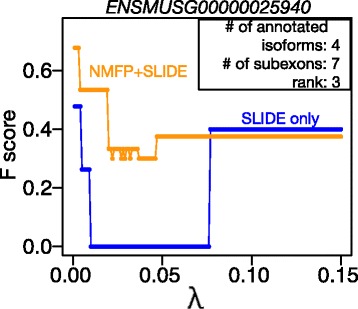
NMFP increases the robustness of SLIDE to the choices of regularization parameter *λ*, from 0.001 to 0.15 by 0.001. F scores of NMFP + SLIDE (*orange*) and SLIDE (*blue*) were calculated at 150 values of *λ* used in the Lasso regression in SLIDE. NMFP used an NMF rank =3. RNA-seq data were simulated from mouse gene *ENSMUSG00000025940*, which has 7 subexons and 4 annotated isoforms

By shrinking the searching space of possible isoforms, NMFP can effectively increase the robustness of SLIDE to its regularization parameter *λ* in the Lasso estimation. We conduct case studies on three mouse genes, for which we apply NMFP + SLIDE and SLIDE with 150 *λ* values, ranging from 0.001 to 0.15 by 0.001. The results show that NMFP + SLIDE has better isoform discovery accuracy than SLIDE for smaller *λ* values (Fig. 6 and Additional file 1: Figure S3). This finding is expected because a smaller search space does not need a large regularization parameter in the Lasso estimation. It indicates that NMFP can reduce the difficulty of choosing *λ* in the use of SLIDE, as users can simply choose a small *λ* value to obtain good isoform discovery results.

#### Robustness of NMFP to the number of samples

**Fig. 7:**
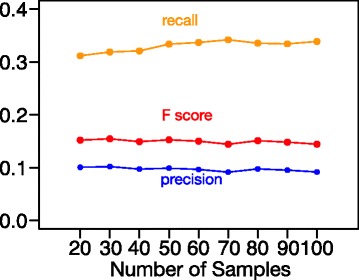
Robustness of NMFP to the number of input RNA-seq samples

In practice, the number of RNA-seq data sets under the same biological condition can be quite small, though this issue has become less of a concern given the rapid accumulation of RNA-seq data sets in public repositories. To make NMFP a practically useful tool, it is important to evaluate its robustness to *m*, the number of input samples in the read count matrix **V**. We apply NMFP to different numbers of simulated mouse samples, from 20 to 100 samples, following the sample order with increasing sequencing depths (for the sample order, please see Section 3.2 in the Additional file 1). As shown in Fig. 7, the NMFP results are robust to the number of samples and differences in sequencing depths.

### Simulation study on the effects of expression levels

**Fig. 8:**
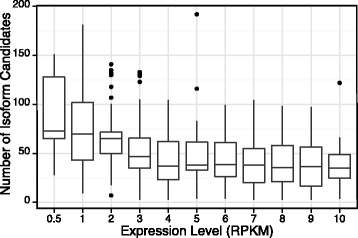
Numbers of isoform candidates found by NMFP to cover true isoforms vs. gene expression levels in additional samples other than the sample of interest

We use another simulation study to demonstrate that NMFP is capable of increasing the isoform discovery accuracy for a lowly expressed gene in a sample by leveraging other samples (Fig. 8). In this simulation, we construct a gene with 8 exons and 3 true isoforms (10111111), (10011111) and (01111111), which indicate the skipping of Exon 2, Exons 2 and 3, and Exon 1 respectively. The exon lengths are randomly sampled from {100,150,200,…,500}. We simulate 10 samples. In each sample, the proportions of the three isoforms are randomly simulated from *U* [ 0,1], and the three proportions are normalized to sum up to 1. Given an isoform, single-end reads with length 76 bp are assumed to follow a uniform distribution on this isoform. We fix the gene expression level in Sample 1 as 3 RPKM (Reads Per Kilobase of transcript per Million mapped reads) and vary the gene expression levels in the other nine samples from 0.5, 1, 2 to 10 RPKM in 11 different settings. In each setting, we calculate a read count matrix **U** from the gene expression levels, the isoform proportions, and the uniformity assumption. Then we add white Gaussian noise *N*(0,1) to **U**, and normalize the resulting matrix as **V**, for which we apply NMFP. Figure 8 depicts the relationship between the gene expression level in Samples 2–10 and the minimum number of isoform candidates found by NMFP to cover the three true isoforms. When the gene expression level is below 3 RPKM, the number of isoform candidates needed to cover the true isoforms is more than 60 with a large variance.

Once the gene expression level goes above 3 RPKM, the number of needed candidates decreases to approximately 30 and has a reduced variance. This result demonstrates that for a gene with low expression in a sample, NMFP can take advantage of other samples to help reduce the number of isoform candidates of this gene. This property makes it possible for downstream isoform discovery methods to explore isoform structures of lowly expressed genes in a sample given the availability of other samples where the genes are more highly expressed.

### Real data case study

We further demonstrate the performance of NMFP and compare the seven methods on 74 real RNA-seq data sets of *D. melanogaster* (reference genome dm5 and annotation BDGP5 of release 66). Description of the 74 data sets is in Section 5 of the Additional file 1. Since we have no knowledge on the true isoforms existing in real RNA-seq data sets, we interpret the results by studying a few genes in Integrative Genomics Viewer (IGV) [24, 25], where we plot the read distribution in an RNA-seq data set (*D. melanogaster* pupae 3 days) and the isoforms identified by the seven methods.

**Fig. 9:**
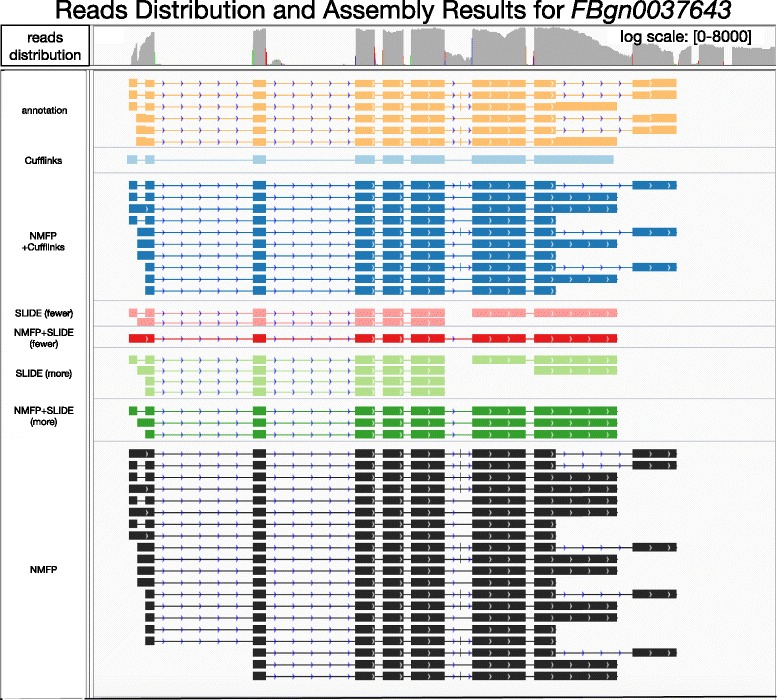
RNA-seq read coverage, annotation and isoform discovery results of *FBgn0037643*

For *D. melanogaster* gene *FBgn0037643*, which has 11 subexons and 6 annotated isoforms, NMFP finds 20 isoform candidates, which contain 4 annotated isoforms and miss the other two by a very short subexon (of only 3 bp). Cufflinks only identifies one annotated isoform, which cannot explain the observed read distribution in the last subexon. In contrast, NMFP + Cufflinks successfully identifies four annotated isoforms, which successfully capture the last subexon. NMFP also helps SLIDE discover full-length annotated isoforms instead of isofrom fragments (Fig. 9). More real data case studies are in Section 3.4 of the Additional file 1.

## Conclusion and discussions

NMF is inherently capable of providing interpretable results for the problem of mRNA isoform discovery. From an input matrix of RNA-seq read counts, NMF can find a sparse structure to indicate which bins are likely to form one isoform. However, NMF alone is not competent for recovering isoforms from RNA-seq data because of several reasons: (i) inaccurate read coverages and missing junction reads due to RNA-seq biases and mapping difficulties, (ii) non-uniqueness of NMF solutions leading to ambiguous isoform discovered, (iii) conflicting bins co-existing in discovered isoforms making the results biologically invalid, and (iv) difficulty in determining the NMF rank. Those issues can be largely alleviated or resolved when NMF is used as a method to pre-select isoform candidates rather than determining final isoforms. For (i), (ii) and (iv), since each non-unique NMF solution provides isoform candidates that may include some true isoforms among many false positives, aggregating multiple NMF solutions to find the high-frequency isoforms will increase the chance of discovering the true isoforms. We demonstrate in the simulation study of *M. musculus*, that the NMFP results are not sensitive to the choice of NMF ranks. We address (iii) by adding a penalty term to NMF and decomposing the invalid isoform candidates with conflicting bins into biologically valid isoforms.

We propose NMFP as a preselection method for improving the accuracy of downstream isoform discovery methods. NMFP takes input from annotated genes and exon boundaries (e.g. UCSC and Ensemble annotations) and/or de novo assemblies (e.g. Cufflinks output). It outputs a small pool of isoform candidates, which are expected to well cover true isoforms and can form a reduced search space for downstream methods. From a general perspective, combination of methods with opposing advantages and drawbacks may improve over individual methods. NMFP and SLIDE are both built on the idea of selecting isoforms from all possibilities, while Cufflinks assembles transcripts directly via an overlap graph. A possible reason for NMFP + Cufflinks to become the top performer is that it combines the strengths of two different approaches: selection and assembly. This may also explain why NMFP + SLIDE, which combines two selection based methods, is outperformed by NMFP + Cufflinks, though SLIDE alone outperforms Cufflinks without NMFP. However compared to SLIDE itself, NMFP + SLIDE still has improved performance at the transcript level, and even becomes more robust to the regularization parameter choices. Overall, we demonstrate by simulation and real data studies that NMFP is a useful tool for improving isoform discovery accuracy and robustness in many aspects.

Nevertheless, NMFP still encounters several problems and there remains substantial room for improvement. The noise and bias generated during RNA-seq still has large influence on NMFP results. Some methods for denosing and debiasing may help the performance of NMFP [26, 27]. In addition, NMFP requires enough samples to implement NMF, for which normalization is important but difficult for samples generated by different platforms or from different sources. Normalization methods for RNA-seq data (e.g., [28]) may improve the performance of NMFP. Moreover, the current NMFP algorithm has high computational complexity, and optimizing the algorithm is one of our top priority. We also intend to build a database of isoform candidates found by NMFP for different species, so that researchers can conveniently use them for downstream analysis without aggregating multiple samples and going through the whole NMFP pipeline.

## Additional file

Additional file 1
**More detail on the methods and results; more simulation and real data results.** (PDF 606 kb)

## Abbreviations

NMF: non-negative matrix factorization
NMFP: non-negative matrix factorization preselection
mRNA: messenger RNA
cDNA: complementary DNA
qPCR: quantitative polymerase chain reaction
