# Erratum to: ‘NMFP: a non-negative matrix factorization based preselection method to increase accuracy of identifying mRNA isoforms from RNA-seq data’

**DOI:** 10.1186/s12864-016-2621-6

**Published:** 2016-04-19

**Authors:** Yuting Ye, Jingyi Jessica Li

**Affiliations:** Division of Biostatistics, University of California, Berkeley, USA; Department of Statistics, University of California, 8125 Math Sciences Bldg, Los Angeles, USA; Department of Human Genetics, University of California, 695 Charles E. Young Drive South, Los Angeles, USA

Unfortunately, the original version of this article [1] contained an error. The authors noted an error only in the HTML version of their article. The equation (8) has a typo in it. It is shown as:

$$ \mathrm{D}\left(\mathrm{V},\backslash,\ \mathrm{W}\ \mathrm{H}\right) $$

However it should be shown as the following:

$$ \mathrm{D}\left(\mathrm{V},\ \mathrm{W}\ \mathrm{H}\right) $$
