# Supplementary material for: NMFP: a non-negative matrix factorization based preselection method to increase accuracy of identifying mRNA isoforms from RNA-seq data
Source: BMC Genomics. 2016 Jan 11;17(Suppl 1):11. doi: 10.1186/s12864-015-2304-8 (PMC4895266; doi:10.1186/s12864-015-2304-8)
Supplement: Additional file 1 — More detail on the methods and results; more simulation and real data results. (PDF 606 kb) [file 12864_2015_2304_MOESM1_ESM.pdf]

Additional File for "A non-negative matrix factorization based preselection procedure for more accurate isoform discovery from RNA-seq data"

Yuting Ye*

Division of Biostatistics, University of California, Berkeley

Jingyi Jessica Li†

Department of Statistics, University of California, Los Angeles

1 Subexons and contradicting bins

1.1 Definition of subexons

Exons are not the minimal splicing units. In some types of alternative splicing, such as alternative 5' ends and alternative 3' ends, splicing can occur inside an exon. Also, there can be differences between the exon boundaries from annotations and those from de novo assembles. Hence to capture slight differences among isoform structures, we split exons into *subexons*, the minimal splicing units. Subexons are defined as non-overlapping transcribed regions between adjacent splicing sites. Every exon in the input annotation or de novo assembly can be fully recovered by a set of subexons. For illustration of subexons, please see Figure 1 extracted from the SLIDE paper[1].

1.2 Contradicting bins

We define *bins* as two-dimensional vectors that describe the exon indices of the starting and ending positions of mapped reads (single-ended reads or paired-end reads decomposed into two ends). For example, Bin $(4, 4)$ contains reads whose starting and ending positions

*Email: yeyt@berkeley.edu
†Email: jli@stat.ucla.edu; Corresponding author

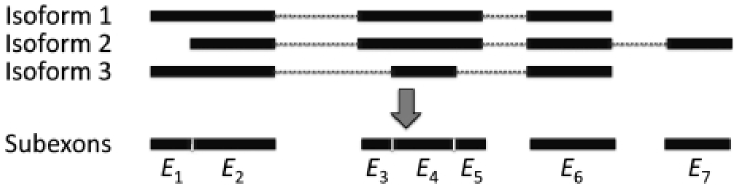

Figure 1: **Definition of the subexon**

are both in Subexon 4. For reads that cannot originate from the same transcript, their corresponding bins are mutually exclusive. We call them *contradicting bins*. For example, Bins $(4, 4)$ and $(3, 5)$ are contradicting bins, because Bin $(4, 4)$ indicates the existence of Subexon 4 but Bin $(3, 5)$ indicates the skipping of Subexon 4.

1.3 Decomposing isoforms candidates containing contradicting bins

After non-negative matrix factorization (NMF) is completed, a basis matrix W would be obtained, and each column of W represents an isoform candidate (See the main text). However, isoform candidates may contain contradicting bins, and such candidates cannot be true isoforms. To resolve this issue without losing possibly true isoforms, we decompose an isoform candidate with two contradicting bins into two isoform candidates, each containing one of the two bins. We use **Figure 1** as an example. Suppose an isoform candidate contains contradicting Bins $(4, 4)$ and $(3, 5)$, which indicate contradicting status of Subexon 4. Suppose all the other bins are non-contradicting and indicate the existence of Subexons 1, 2, 3, 5, 6, and 7. Then we decompose the isoform candidate into two candidates: 1111111 and 1110111, where the former contains all subexons and supports Bin $(4, 4)$ while the latter excludes Subexon 4 and supports Bin $(3, 5)$. This procedure is to reduce our chance of missing true isoforms.

2 K-means and gap statistic

2.1 Motivation

With objective function $\min_{W \geq 0, H \geq 0} ||V - WH||_F$ and additional orthogonality constraint on H, i.e., $H^T H = I$, NMF can be regarded as one type of K-means clustering on the bins (rows of V) with non-negativity constraint. The reason is that the purpose of NMF is to cluster bins into *bin groups*, which are sub-structures of isoforms and can form into multiple isoforms including the true ones. This motivated us to use the *gap statistic*, a method for choosing the number of cluster K in K-means clustering, to select the rank of NMF. Gap

statistic was proposed by Tibshirani et al. [2] and has since been a widely used metric for choosing K in K-means clustering because of its good performance in estimating the number of well separated clusters.

2.2 K-means clustering

Suppose there are n p-dimensional data points, $X_1, X_2, \ldots, X_n \in \mathbb{R}^p$ and the goal is to cluster them into K clusters $C_1, \ldots, C_K$. Given K, K-means clustering would assign the n data points to K clusters, i.e., find the cluster memberships $C_1, \ldots, C_K$ by minimizing the following objective function

$$\arg \min_{C_1,\ldots,C_k} \sum_{r=1}^{K} \sum_{i \in C_r} ||X_i - \mu_r||, \tag{1}$$

where μ_r is the mean of cluster C_r, which is a subset of the n data points. Formula (1) is equivalent to

$$\arg \min_{C_1,\ldots,C_k} \sum_{r=1}^{K} \frac{1}{2n_r} \sum_{i,j \in C_r} d_{ij} \tag{2}$$

n_r is the number of points in cluster C_r and d_{ij} is the distance between X_i and X_j, i.e. $||X_i - X_j||$. There are many choices for the distance metric, such as the Euclidean distance. The objective funciton $W_K = \sum_{r=1}^{K} \frac{1}{2n_r} \sum_{i,j \in C_r} d_{ij}$ is the within-cluster variance, which is a basic statistic for determining K.

2.3 Gap statistic

Gap statistic is defined as $Gap_n(k) = E_k^*[\log(W_k)] - \log(W_k)$. The first term is the expected W_k under a reference distribution with no clusters, and the second term is the observed W_k. The idea is to choose the number of clusters as the value of k that leads to the largest $Gap_n(k)$. To estimate $E_k^*[\log(W_k)]$, the simplest reference distribution is the uniform distribution in all the p dimensions over the range of the observed data. The gap statistic algorithm sketched below is from the original gap statistics paper [2].

1. Vary the number of clusters $k = 1, \ldots, T$, and cluster the data $X_1, \ldots, X_n$ by K-means clustering into k clusters, resulting in W_k, $k = 1, \ldots, T$, where T is the upper bound on k.

2. Generate B reference data sets from the specified reference distribution (e.g. uniform distribution). Then we cluster each data set into k clusters, resulting in W_{kb}^*, $k = 1, \ldots, T$; $b = 1, \ldots, B$.

3. Let $\bar{w} = \frac{1}{B} \sum_{b=1}^{B} \log(W_{kb}^*)$, $sd_k = \sqrt{\frac{1}{B} \sum_{b=1}^{B} (\log(W_{kb}^*) - \bar{w})^2}$, $s_k = sd_k \sqrt{1 + \frac{1}{B}}$.

4. Estimate the gap statistic as $\hat{Gap}_n(k) = \bar{w} - log(W_k)$, for $k = 1, \ldots, T$.

5. Choose the number of clusters as $\hat{K}$ = smallest k s.t. $\hat{Gap}_n(k) \geq \hat{Gap}_n(k+1) - s_{k+1}$.

2.4 Application of gap statistic to NMF rank determination

NMF is a way of K-means clustering that clusters the bins with similar expression levels into the bin groups, i.e., splicing structures that can be reconstructed into isoforms. In most cases, the number of bin groups is close to the number of isoforms. For exmple, assume there is a 5-subexon gene with 3 isoform, 11111, 11011 and 11101. The relative abundance of the three isoforms are 50%, 35% and 15% respectively. Then the subexons have relative expression levels as 100%, 100%, 65%, 85% and 100% sequentially. Therefore, Subexons 1, 2 and 5 will be clustered into one bin group, while Subexon 3 and Subexon 4 will each be clustered as one bin group respectively. In this example, both the number of isoforms and the number of bin groups are 3. For genes with more complicated splicing structures, the number of bin groups may be more than the number of isoforms. In such cases, our estimated number of bin groups, $\hat{K}$ from gap statistic, could be larger than the number of true isoforms. However, from our simulation results, we observed that NMFP is not sensitive to the NMF rank choice and performs reasonably well as long as the rank is no less than the number of annotated isoforms. (See the section **Low sensitivity of NMFP to ranks** in the main text.) Combined with the fact that gap statistic tends to be conservative [2], the NMF rank should be better chosen as larger than $\hat{K}$, the number of clusters chosen by the gap statistic on V. In our results, we chose the NMF rank as $\hat{K} + 1$.

3 More results

3.1 Robustness of NMFP to the choices of NMF rank (More results)

Continued from the main text, here we attach two more simulation examples to illustrate that NMFP is not sensitive to the choice of NMF rank. In **Figure 2(a)**, Gene *FBgn0259821* has three annotated isoforms (Ensemble BDGP6 of release 80) with 13 subexons. NMFP is able to capture all the annotated isoforms (recall rate = 1) regardless of the rank choices. The precision rate of NMFP is 1 when the rank equals 2. Although it decreases when the rank increases to 3 because higher ranks would lead to more isoform candidates, it becomes relatively stable after rank equals 4. In **Figure 2(b)**, Gene *FBgn0037215* has 5 annotated isoforms with 12 subexons. NMFP has stable performance across all the rank choices.

3.2 Detailed information of genes on chromosome chr1 of *Mus musculus*

In the section **Simulation results in *Mus musculus*** in the main text, we did another simulation to demonstrate the performance of NMFP on mouse transcriptome. Apart

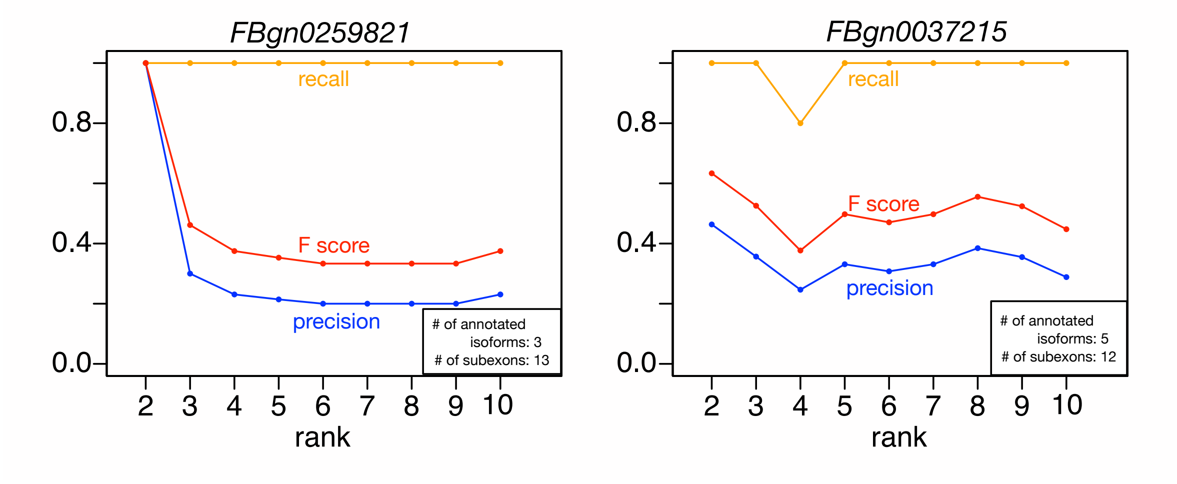

(a) Gene *FBgn0259821*

(b) Gene *FBgn0037215*

Figure 2: **The performance of NMFP in terms of the change of ranks** The orange line represents recall curve, the red line F score curve and the blue line represents precision curve.

from what has been already stated in the main text, some supplementary detail (**Table 2**) is provided here about the genes we selected to work on from chromosome chr1 of *Mus musculus* (reference genome mm10 and annotation GRCm38 of release 81). Here, a brief description is given as following about the parameters for *flux simulator* to simulate the 100 samples. The 100 samples are equally split into 10 groups, *Group* 1 (Sample 1, Sample 2, . . . , Sample 10), *Group* 2 (Sample 11, Sample 12, . . . , Sample 20), . . . , *Group* 10 (Sample 91, Sample 92, . . . , Sample 100). For *Group* i, NB_MOLECULES for *Expression* step is $(20 + i) \cdot 200000$. For the $(10 \cdot (i - 1) + j)_{th}$ sample within *Group* i, READ_NUMBER for *Sequencing* step is $(10 + j) \cdot 1000000$ (**Table 1**). All the samples share other parameters such as that they all use paired-end reads with length of 2×76 bp.

3.3 Increased robustness of NMFP+SLIDE to the choices of parameter λ (More results)

Continued from the main text, here we include two more simulation results to show that NMFP can help SLIDE achieve better isoform discovery accuracy at lower values of λ, the regularization parameter used in the LASSO step in SLIDE. Hence, the choice of a proper value for λ becomes an easier task for SLIDE+NMFP than for SLIDE. In **Figure 3 (a)**, Gene *ENSMUSG00000025905* has 5 annotated isoforms with 12 subexons. The rank is set

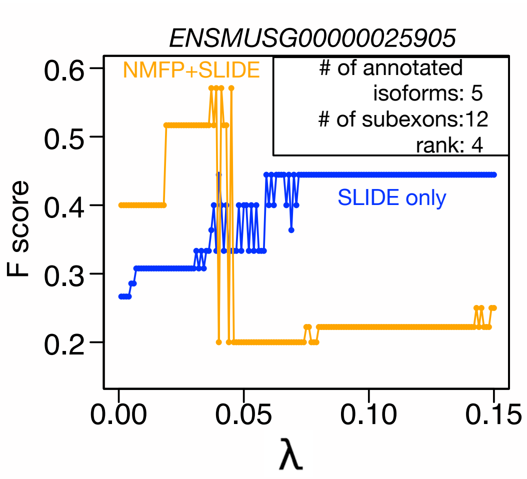

(a) Gene *ENSMUSG00000025905*

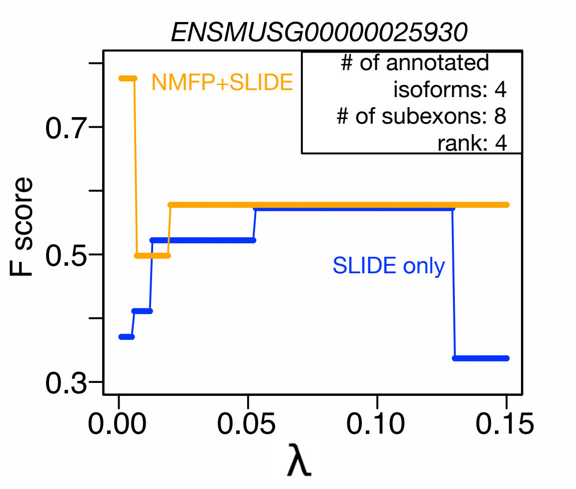

(b) Gene *ENSMUSG00000025930*

Figure 3: **The performance of NMFP+SLIDE vs. SLIDE at various λ values.** The orange line represents the F scores of NMFP+SLIDE, while the blue line represents the F scores of SLIDE alone.

as 4. NMFP+SLIDE has much higher F scores than SLIDE for $\lambda < 0.04$. In **Figure 3 (b)**, Gene *ENSMUSG00000025930* has 4 annotated isoforms with 8 subexons. The NMF rank is set as 4. We also observe that NMFP+SLIDE has better performance than SLIDE especially when $\lambda < 0.015$. Since NMFP can largely reduce the isoform candidate pool for SLIDE, it is recommended to use a small λ value for NMFP+SLIDE.

Table 1: **Parameters for the 100 simulated samples of *Mus musculus***

	Sample 1	Sample 2	...	Sample 10	Sample 11	...	Sample 20	...
NB_MOLECULES	4,200,000	4,200,000	...	4,200,000	4,400,000	...	4,400,000	...
READ_NUMBER	11,000,000	12,000,000	...	20,000,000	11,000,000	...	20,000,000	...

3.4 Real data case study (More results)

Continued from the main text, we use another two cases to show that NMFP has good performance on real data. In **Figure 4**, RNA-seq reads for gene *FBgn0019936* were generated by the modENCODE consortium [3] from *D. melanogaster* L3 stage larvae and 12 hours post-molt (SRA accession: SRS004682; see the Supplemental Material "Updated Table S2.xlsx" in [4] for more details). This gene has 1 annotated isoform (shown in orange), which is well supported by the RNA-seq reads (shown in gray). Cufflinks alone connected the latter three exons together with the introns in between into one piece (shown in light blue). NMFP+Cufflinks accurately assembled the annotated isoform and recovered another isoform (shown in dark blue), which reflects the low read counts of the Exon 3. Similarly, NMFP+SLIDE at $\lambda = 0.2$ ("more", shown in dark green) and $\lambda = 0.01$ ("fewer", shown in dark red) achieved better isoform discovery results than their SLIDE counterparts (shown in light green and light red). In **Figure 5**, RNA-seq reads for gene *FBgn0038145* were also generated by the modENCODE corsortium from the heads of mated female *D.melanogaste* after 1 day of eclosion (SRA accession: SRR070434, SRR070435 andSRR100279; see the Supplemental Material "Updated Table S2.xlsx" in [4] for more details). *FBgn0038145* has a complicated splicing structure and 5 annotated isoforms (shown in orange). Cufflinks alone assembled one transcript (shown in light blue) similar to the first annotated one except that part of Exon 1 is missed. NMFP+Cufflinks identified 4 isoforms (shown in dark blue) among which 2 are annotated. NMFP also improved the performance of SLIDE at both $\lambda = 0.2$ ("more", shown in light and dark green) and $\lambda = 0.01$ ("fewer", shown in light and dark red). One significant contribution of NMFP to Cufflinks and SLIDE is capturing Exon 2, which is missed by Cufflinks and SLIDE alone because of its low read coverage compared to the other exons.

Table 2: **Summary of the genes used in the section "Simulation results in *Mus musculus*" in the main text.** The table lists the numbers of the genes that have 3-30 subexons and 2-17 annotated isoforms.

# of subexons n	$3 \leq n \leq 6$	$7 \leq n \leq 10$	$11 \leq n \leq 14$	$15 \leq n \leq 18$	$19 \leq n \leq 22$	$n \geq 23$
	155	185	163	134	89	126
# of isoforms q	$2 \leq q \leq 3$	$4 \leq q \leq 5$	$6 \leq q \leq 7$	$8 \leq q \leq 9$	$10 \leq q \leq 11$	$q \geq 12$
	382	206	133	81	22	28

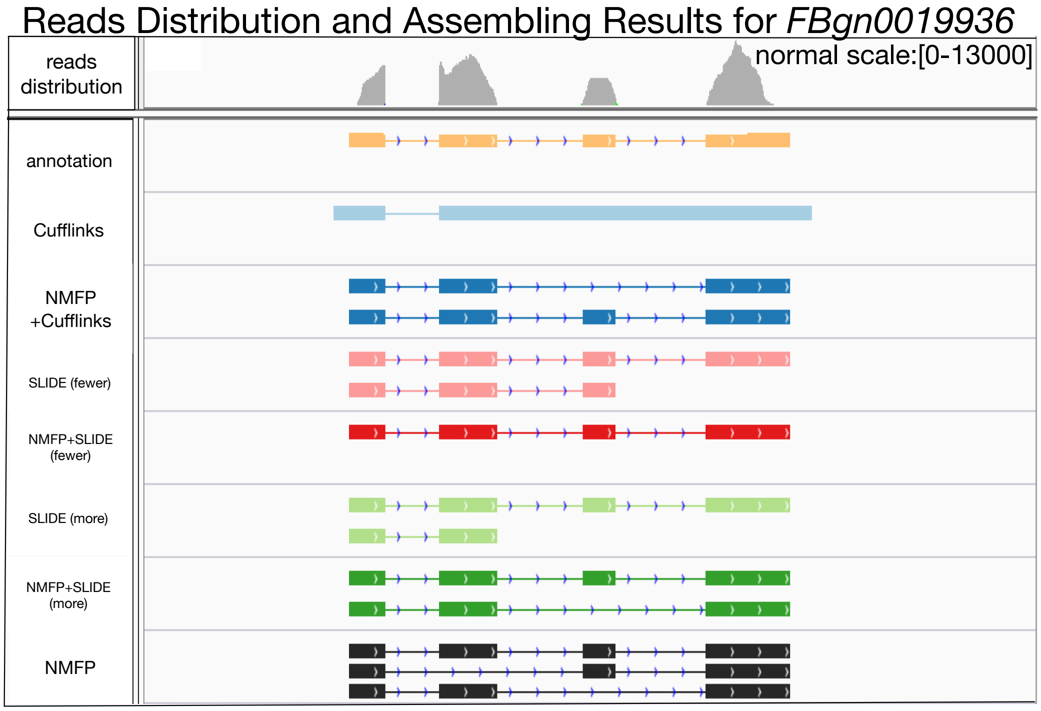

Figure 4: **Real data results for Gene *FBgn0019936***

4 Parameters for Flux Simulator

In this Section, we append the detailed contents of the parameter file for Sample 1 in "Simulation results in *D.melanogaster*" of the main text. The parameter file is required for *flux simulator* to simulate RNAseq reads. Note that REF_FILE_NAME is the name for the annotation file (chr3R part of annotation Ensembl BDGP6 of release 80) and GEN_DIR is a directory to store its corresponding genome files (genome dm6). Please refer to Flux Simulator for more details.

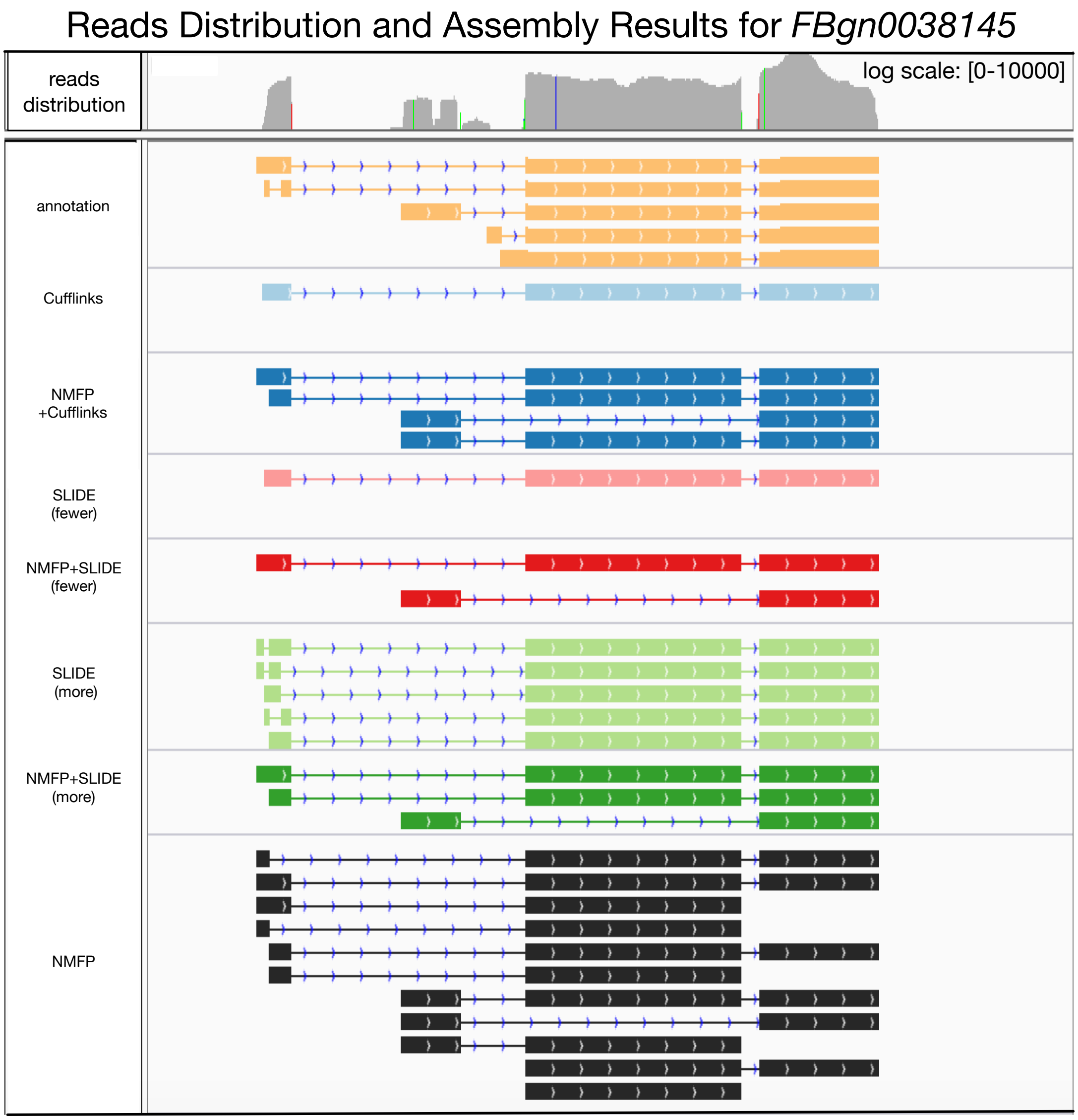

Figure 5: **Real data results for Gene *FBgn0038145***

```
##################  The parameter file for Sample 1 ####################
## File locations
REF_FILE_NAME   chr3R_BDGP6.gtf
GEN_DIR         chromFa/

## Expression
NB_MOLECULES    1000000
TSS_MEAN 25
POLYA_SCALE     300
POLYA_SHAPE     2
EXPRESSION_X0   9500
EXPRESSION_X1   90250000

## Fragmentation
FRAG_SUBSTRATE  DNA
FRAG_METHOD     UR
FRAG_UR_ETA     300
FRAG_UR_D0      76

## RT parameters
RTRANSCRIPTION YES
RT_MOTIF       default
RT_PRIMER      RH
RT_LOSSLESS    YES
RT_MIN         500
RT_MAX         5500

## PCR / Filtering
PCR_DISTRIBUTION  default
FILTERING         YES
SIZE_DISTRIBUTION N(300,74)
SIZE_SAMPLING     AC

# Sequencing
READ_NUMBER     5000000
READ_LENGTH     76
PAIRED_END      YES
FASTA YES
ERR_FILE 76
UNIQUE_IDS YES
```

5 Summary of 74 real data samples

Tables 3-10 summarize the description of the 74 *D. melanogaster* RNA-seq data sets [3, 4] we use in our real data case study.

References

[1] Li, J.J., Jiang, C.-R., Brown, J.B., Huang, H., Bickel, P.J.: Sparse linear modeling of next-generation mrna sequencing (rna-seq) data for isoform discovery and abundance estimation. Proceedings of the National Academy of Sciences **108**(50), 19867–19872 (2011)

[2] Tibshirani, R., Walther, G., Hastie, T.: Estimating the number of clusters in a data set via the gap statistic. Journal of the Royal Statistical Society: Series B (Statistical Methodology) **63**(2), 411–423 (2001)

[3] Gerstein, M.B., Rozowsky, J., Yan, K.-K., Wang, D., Cheng, C., Brown, J.B., Davis, C.A., Hillier, L., Sisu, C., Li, J.J., *et al.*: Comparative analysis of the transcriptome across distant species. Nature **512**(7515), 445–448 (2014)

[4] Li, J.J., Huang, H., Bickel, P.J., Brenner, S.E.: Comparison of d. melanogaster and c. elegans developmental stages, tissues, and cells by modencode rna-seq data. Genome research **24**(7), 1086–1101 (2014)

Table 3: **Summary of RNA-seq reads from 74 real data samples for *D.melanogaster* (Part I).** All these reads were generated from *Illumina* RNA-seq.

Sample Name	Embryo0-2h	Embryo2-4h	Embryo4-6h	Embryo6-8h	Embryo8-10h
SRA accession	SRS004668	SRS004669	SRS004670	SRS004671	SRS004672
Sample Description	Embyros, 0-2 hour after egg laying	Embyros, 2-4 hour after egg laying	Embyros, 4-6 hour after egg laying	Embyros, 6-8 hours after egg laying	Embyros, 8-10 hours after egg laying
Organ/Tissue	Whole organism	Whole organism	Whole organism	Whole organism	Whole organism
Age	Embryo	Embryo	Embryo	Embryo	Embryo
Sex	Mixed Male/Female	Mixed Male/Female	Mixed Male/Female	Mixed Male/Female	Mixed Male/Female
Single- vs paired-end	paired	paired	paired	paired	paired
Reads sequenced(M)	101	80	193	119	108

Sample Name	Embryo10-12h	Embryo12-14h	Embryo14-16h	Embryo16-18h	Embryo18-20h
SRA accession	SRS004673	SRS004674	SRS004675	SRS004676	SRS004677
Sample Description	Embyros, 10-12 hour after egg laying	Embyros, 12-14 hour after egg laying	Embyros, 14-16 hour after egg laying	Embyros, 16-18 hours after egg laying	Embyros, 18-20 hours after egg laying
Organ/Tissue	Whole organism	Whole organism	Whole organism	Whole organism	Whole organism
Age	Embryo	Embryo	Embryo	Embryo	Embryo
Sex	Mixed Male/Female	Mixed Male/Female	Mixed Male/Female	Mixed Male/Female	Mixed Male/Female
Single- vs paired-end	paired	paired	paired	paired	paired
Reads sequenced(M)	139	180	137	117	127

Table 4: **Summary of RNA-seq reads from 74 real data samples for *D.melanogaster* (Part II).** All these reads were generated from *Illumina* RNA-seq.

Sample Name	Embryo20-22h	Embryo22-24h	L1	L2	L3+12h
SRA accession	SRS004678	SRS004679	SRS004680	SRS004681	SRS004682
Sample Description	Embyros, 20-22 hour after egg laying	Embyros,22-24 hour after egg laying	Embyros, L1 stage larvae	L2 stage larvae	L3 stage larvae, 12 hr post-molt
Organ/Tissue	Whole organism	Whole organism	Whole organism	Whole organism	Whole organism
Age	Embryo	Embryo	L1	L2	L3
Sex	Mixed Male/Female	Mixed Male/Female	Mixed Male/Female	Mixed Male/Female	Mixed Male/Female
Single- vs paired-end	paired	paired	paired	paired	paired
Reads sequenced(M)	74	155	126	189	73
Sample Name	L3PS1-2	L3PS3-6	L3PS7-9	Prepupae	Prepupae+12h
SRA accession	SRS004686	SRS004687	SRS004867	SRS004668	SRS004701
Sample Description	L3 stage larvae, dark blue gut, puff stage 1-2	L3 stage larvae, light blue gut, puff stage 3-6	L3 stage larvae, clear gut puff stage 7-9	White prepupae	Pupae, 12 hours after white prepupae
Organ/Tissue	Whole organism	Whole organism	Whole organism	Whole organism	Whole organism
Age	L4	L5	L6	Pupa	Pupa
Sex	Mixed Male/Female	Mixed Male/Female	Mixed Male/Female	Mixed Male/Female	Mixed Male/Female
Single- vs paired-end	paired	paired	paired	paired	paired
Reads sequenced(M)	80	73	103	114	129

Table 5: **Summary of RNA-seq reads from 74 real data samples for *D.melanogaster* (Part III).** All these reads were generated from *Illumina* RNA-seq.

Sample Name	Prepupae+24h	Prepupae+2d	Prepupae+3d	Prepupae+4d	Male+1d
SRA accession	SRS004702	SRS004869	SRS004870	SRS004703	SRS004695
Sample Description	Pupae, 24 hours after white prepupae	Pupae, 2 days after white prepupae	Pupae, 3 days after white prepupae	Pupae, 4 days after white prepupae	Adult male, one day after eclosion
Organ/Tissue	Whole organism	Whole organism	Whole organism	Whole organism	Whole organism
Age	Pupa	Pupa	Pupa	Pupa	Adult
Sex	Mixed Male/Female	Mixed Male/Female	Mixed Male/Female	Mixed Male/Female	Male
Single- vs paired-end	paired	paired	paired	paired	paired
Reads sequenced(M)	105	117	154	106	112
Sample Name	Male+5d	Male+30d	Female+1d	Female+5d	Female+30d
SRA accession	SRS004696	SRS004697	SRS004689	SRS004693	SRS004692
Sample Description	Adult male, 5 days after eclosion	Adult male, 30 days after eclosion	Adult female, one day after eclosion	Adult female, 5 days after eclosion	Adult female, 30 days after eclosion
Organ/Tissue	Whole organism	Whole organism	Whole organism	Whole organism	Whole organism
Age	Adult	Adult	Adult	Adult	Adult
Sex	Male	Male	Female	Female	Female
Single- vs paired-end	paired	paired	paired	paired	paired
Reads sequenced(M)	123	104	122	91	90

Table 6: **Summary of RNA-seq reads from 74 real data samples for *D.melanogaster* (Part IV).** All these reads were generated from *Illumina* RNA-seq.

Sample Name	CarcassL3	CarcassMixed MaleFemale +1d	CarcassMixed MaleFemale +4d	CarcassMixed MaleFemale +20d	FatL3
SRA accession	SRR100269, SRR070426	SRR070395, SRR070399	SRR070387, SRR070402	SRR070391, SRR070404	SRR070405, SRR070406
Sample Description	third instar larvae, wandering stage, carcass	mixed males and females, eclosion + 1 day, carcass	mixed males and females, eclosion + 4 days, carcass	mixed males and females, eclosion + 20 days, carcass	third instar larvae, wandering stage, fat body
Organ/Tissue	Muscle	Muscle	Muscle	Muscle	Endocrine/Liver
Age	L3	Adult	Adult	Adult	L3
Sex	Mixed Male/Female	Mixed Male/Female	Mixed Male/Female	Mixed Male/Female	Mixed Male/Female
Single- vs paired-end	paired	paired	paired	paired	paired
Reads sequenced(M)	88	115	92	64	61

Sample Name	FatPrepupae	FatPrepupae FatPrepupae	SalivaryGlands L3	SalivaryGlands Prepupae	DigestiveSystem L3
SRA accession	SRR070411, SRR070428	SRR070429, SRR070413	SRR070425, SRR070407	SRR070427, SRR100270	SRR100268, SRR070408
Sample Description	white prepupae, fat body	pupae, white prepupae+2d, fat	third instar larvae, wandering stage, salivary glands	white prepupae, salivary glands	third instar larvae, wandering stage, digestive system
Organ/Tissue	Endocrine/Liver	Endocrine/Liver	Exocrine gland	Exocrine gland	Gut
Age	L4/Pupa	L4/Pupa	L3	L4/Pupa	L3
Sex	Mixed Male/Female	Mixed Male/Female	Mixed Male/Female	Mixed Male/Female	Mixed Male/Female
Single- vs paired-end	paired	paired	paired	paired	paired
Reads sequenced(M)	93	55	83	94	94

Table 7: **Summary of RNA-seq reads from 74 real data samples for *D.melanogaster* (Part V).** All these reads were generated from *Illumina* RNA-seq.

Sample Name	DigestiveSystem MixedMaleFemale+1d	DigestiveSystem MixedMaleFemale+4d	DigestiveSystem MixedMaleFemale+20d	ImaginalDiscsL3	CNSL3
SRA accession	SRR070394, SRR070398	SRR070401, SRR070386, SRR111878, SRR111879	SRR070403, SRR070390, SRR111883	SRR070392, SRR111884, SRR350962, SRR070393, SRR111885, SRR350963	SRR070409, SRR070410
Sample Description	mixed males and females, eclosion + 1 day, digestive system	mixed males and females, eclosion + 4 days, digestive system	mixed males and females, eclosion + 20 days, digestive system	third instar larvae, wandering stage, imaginal discs	third instar larvae, CNS
Organ/Tissue	Gut	Gut	Gut	Epithelial	Neural
Age	Adult	Adult	Adult	L3	L3
Sex	Mixed Male/Female	Mixed Male/Female	Mixed Male/Female	Mixed Male/Female	Mixed Male/Female
Single- vs paired-end	paired	paired	paired	paired	paired
Reads sequenced(M)	60	165	106	415	62
Sample Name	CNSPrepupae +2d	HeadsVirgin Female+1d	HeadsVirgin Female+4d	HeadsVirgin Female+20d	HeadsMated Female+1d
SRA accession	SRR100271, SRR070412	SRR070436, SRR070437, SRR100281	SRR070430, SRR100278, SRR100282	SRR070388, SRR070419, SRR100275	SRR070434, SRR070435, SRR100279
Sample Description	pupae, white prepupae+2d, CNS	virgin female, eclosion + 1 day, heads	virgin female, eclosion + 4 days, heads	virgin female, eclosion + 20 days, heads	mated female, eclosion + 1 day, heads
Organ/Tissue	Neural	Neural	Neural	Neural	Neural
Age	L4/Pupa	Adult	Adult	Adult	Adult
Sex	Mixed Male/Female	Female	Female	Female	Female
Single- vs paired-end	paired	paired	paired	paired	paired
Reads sequenced(M)	104	278	253	114	264

Table 8: **Summary of RNA-seq reads from 74 real data samples for *D.melanogaster* (Part VI).** All these reads were generated from *Illumina* RNA-seq.

Sample Name	HeadsMated Female+4d	HeadsMated Female+20d	HeadsMated Male+1d	IHeadsMated Male+4d	HeadsMated Male+20d
SRA accession	SRR070414, SRR070415	SRR116383, SRR111882, SRR070420, SRR100274	SRR070432, SRR070433, SRR100280	SRR070416, SRR070400	SRR070421, SRR070424
Sample Description	mated female, eclosion + 4 days, heads	mated female, eclosion + 20 days, heads	mated male, eclosion + 1 day, heads	mated male, eclosion + 4 day, heads	mated male, eclosion + 20 day, heads
Organ/Tissue	Neural	Neural	Neural	Neural	Neural
Age	Adult	Adult	Adult	Adult	Adult
Sex	Female	Female	Male	Male	Male
Single- vs paired-end	paired	paired	paired	paired	paired
Reads sequenced(M)	138	71	196	127	105
Sample Name	OvariesVirgin Female+4d	OvariesMated Female+4d	TestesMated Male+4d	AccessoryGlands Mated-Male+4d	(Embryo) GM2
SRA accession	SRR070396, SRR070417	SRR070431, SRR100277, SRR100283	SRR070422, SRR350960, SRR070423, SRR100276, SRR350961	SRR070397, SRR111880, SRR182357, SRR070418, SRR100272, SRR100273, SRR111881, SRR182358, SRR350959	SRR070278, SRR070265, SRR070263
Sample Description	virgin female, eclosion + 4 days, ovaries	mated female, eclosion + 4 days, ovaries	mated male, eclosion + 4 days, testes	mated male, eclosion + 4 days, accessory glands	cell line GM2 from embryos
Organ/Tissue	Gonad	Gonad	Gonad	N.D.	N.D.
Age	Adult	Adult	Adult	Adult	Embryo
Sex	Female	Female	Male	Male	N.D.
Single- vs paired-end	paired	paired	paired	paired	single/paired
Reads sequenced(M)	115	273	473	107	65

Table 9: **Summary of RNA-seq reads from 74 real data samples for *D.melanogaster* (Part VII).** All these reads were generated from *Illumina* RNA-seq.

Sample Name	(Embryo) Kc167	(Embryo) S1	(Embryo) S2_R_plus	(Embryo) S3	(L3 prothoracic leg disc) CME_L1
SRA accession	SRR070261, SRR070269, SRR111873, SRR070292	SRR070280, SRR070286, SRR111877	SRR1197280	SRR070259, SRR111872, SRR189834, SRR189835	SRR070282, SRR070288
Sample Description	cell line Kc167 from embryos	cell line S1 from embryos	cell line S2 from embryos	cell line S3 from embryos	cell line CME_L1 from L3 larva prothoracic leg discs
Organ/Tissue	N.D.	N.D.	N.D.	N.D.	ventral prothoracic disc
Age	Embryo	Embryo	Embryo	Embryo	L3
Sex	Female	N.D.	N.D.	N.D.	N.D.
Single- vs paired-end	single/paired	single/paired	single/paired	single/paired	single/paired
Reads sequenced(M)	56	48	278	132	84
Sample Name	(L3 eye-antennal disc) ML-DmD11	(L3 wing disc) CME_W2	(L3 wing disc) ML-DmD16-c3	(L3 wing disc) ML-DmD20-c5	(L3 wing disc) ML-DmD32
SRA accession	SRR070284, SRR070290, SRR111874	SRR070260, SRR070268, SRR111868	SRR070283, SRR070289	SRR1197396	SRR070281, SRR070287, SRR111875
Sample Description	cell line ML-DmD11 from L3 larva eye-antennal discs	cell line CME_W2 from L3 larva wing discs	cell line ML-DmD16-c3 from L3 larva wing discs	cell line ML-DmD20-c5 from L3 larva wing discs	cell line ML-DmD32 from L3 larva wing discs
Organ/Tissue	eye-antennal disc	dorsal mesothoracic disc	dorsal mesothoracic disc	dorsal mesothoracic disc	dorsal mesothoracic disc
Age	L3	L3	L3	L3	L3
Sex	N.D.	N.D.	N.D.	N.D	N.D.
Single- vs paired-end	single/paired	single/paired	single/paired	single/paired	single/paired
Reads sequenced(M)	43	77	56	43	38

Table 10: **Summary of RNA-seq reads from 74 real data samples for *D.melanogaster* (Part VIII).** All these reads were generated from *Illumina* RNA-seq.

Sample Name	(L3 haltere disc) ML-DmD17-c3	(L3 mixed imaginal discs) ML-DmD4-c1	(L3 CNS) ML-DmBG2-c2	(Tumorous blood cells) MBN2
SRA accession	SRR070285, SRR070291	SRR070273, SRR111876	SRR070262, SRR070270, SRR111870	SRR070258, SRR111869, SRR189833
Sample Description	cell line ML-DmD17-c3 from L3 larva haltere discs	cell line ML-DmD4-c1 from L3 larva mixed imaginal discs	cell line ML-DmBG2-c2 from L3 larva CNS	cell line MBN2 from tumorous blood cells
Organ/Tissue	dorsal metathoracic disc	imaginal disc	central nervous system	larval circulatory system - tumorous blood cell
Age	L3	L3	L3	L3
Sex	N.D.	N.D.	N.D.	N.D.
Single- vs paired-end	single/paired	paired	single/paired	single/paired
Reads sequenced(M)	87	116	35	75
